# Are age-related deficits in route learning related to control of visual attention?

**DOI:** 10.1007/s00426-019-01159-5

**Published:** 2019-03-08

**Authors:** Christopher Hilton, Sebastien Miellet, Timothy J. Slattery, Jan Wiener

**Affiliations:** 1 Department of Psychology, Bournemouth University, Poole House, Talbot Campus, Fern Barrow, Poole, Dorset BH12 5BB UK; 2 Active Vision Lab, School of Psychology, University of Wollongong, Northfields Ave, Wollongong, NSW 2522 Australia

## Abstract

Typically aged adults show reduced ability to learn a route compared to younger adults. In this experiment, we investigate the role of visual attention through eye-tracking and engagement of attentional resources in age-related route learning deficits. Participants were shown a route through a realistic virtual environment before being tested on their route knowledge. Younger and older adults were compared on their gaze behaviour during route learning and on their reaction time to a secondary probe task as a measure of attentional engagement. Behavioural results show a performance deficit in route knowledge for older adults compared to younger adults, which is consistent with previous research. We replicated previous findings showing that reaction times to the secondary probe task were longer at decision points than non-decision points, indicating stronger attentional engagement at navigationally relevant locations. However, we found no differences in attentional engagement and no differences for a range of gaze measures between age groups. We conclude that age-related changes in route learning ability are not reflected in changes in control of visual attention or regulation of attentional engagement.

**Electronic supplementary material:**

The online version of this article (10.1007/s00426-019-01159-5) contains supplementary material, which is available to authorized users.

## Introduction

Learning and recalling routes through complex environments is a common task which is essential in maintaining independence in everyday life. Typically aged adults often report difficulty with general navigation (Burns, 1999) and show a reduced ability to learn and recall routes (Head & Isom, 2010; Moffat, Zonderman & Resnick, 2001), to retrace a route backwards (Wiener, Kmecova & de Condappa, 2012), to understand the layout of a known intersection when approaching it from a novel direction (Wiener, de Condappa, Harris & Wolbers, 2013), to bind landmarks to specific locations (Newman & Kazniak, 2000; Head & Isom, 2010) and to learn the sequence of turns along a route (O’Malley, Innes & Wiener, 2018). Older adults also shift preference away from allocentric navigation strategies and use egocentric strategies more than younger adults (Rodgers, Sindone & Moffat, 2012). Age-related navigation deficits are more pronounced in unfamiliar than in familiar environments (Devlin, 2001) and typically become apparent in adults aged between 60 and 69 years (Barrash, 1994). The current explanations of age-related decline in route learning ability focus on neurodegeneration of structures related to stimulus-response-based, egocentric navigation, such as the caudate (see Lester, Moffat, Wiener, Barnes & Wolbers, 2017). In contrast, the roles played by other cognitive domains in age-related route learning declines have received little attention (for a review see Klencklen, Després & Dufour, 2012). In this study, we investigated whether control of visual attention and attentional engagement also contribute to age-related declines in route learning.

Visual information is a vital input for successful navigation (see Ekstom, 2015), particularly in route navigation, where strategies rely heavily on visual cues (Waller & Lippa, 2007). At decision points, for example, gaze is directed towards the eventually chosen path and to specific geometric features such as long lines of sight or changes in geometry (Wiener, Hölscher, Büchner & Konieczny, 2012). In environments with landmarks that are easily identified, the selection and encoding of relevant landmarks is reflected in gaze behaviour (Hamid, Stankiewicz & Hayhoe, 2010; Wenczel, Hepperle & von Stülpnagel, 2017; de Condappa & Wiener, 2014). While these studies demonstrate that gaze behaviour is a measure which is sensitive to behaviour during route learning, so far no study has addressed the question of whether age-related differences in route learning abilities are reflected in differences in gaze behaviour.

Systematic differences in gaze behaviour between younger and older adults have been reported in non-route learning tasks. Dowiasch, Marx, Einhäuser and Bremmer (2015) measured several gaze parameters, whilst older and younger participants walked through an environment. While participants did not solve a specific navigation task, older adults showed reduced saccade frequency, amplitude, peak and average velocity. This is in line with a driving study (Maltz & Shinar, 1999) in which older adults have been reported to make shorter saccades and more fixations, although fixation durations remained the same as in younger adults. This work also reports that when assessing a spatial scene, older adults focus on smaller sub-regions of the stimuli and are less exploratory than younger adults. Similarly, during locomotion, older adults focused on lower portions of the visual scene and to areas closer to themselves in an effort to reduce task error (see Uiga, Cheng, Wilson, Masters & Capio, 2015). These studies include tasks which are not the focus of this experiment, such as locomotion, but they provide some insight into how cognitive ageing affects gaze behaviour.

Control of visual attention is part of the executive function network (Diamond, 2013), which is known to undergo age-related decline, often characterised by working memory deficits (Klencklen, Lavenex, Brandner & Lavenex, 2017). The ageing brain, however, shows increased activation of the prefrontal cortex across both hemispheres (Cabeza, 2002) as a compensatory mechanism to complete executive functioning tasks (Kirova, Bays & Lagalwar, 2015). Dorsal frontal regions have also been implicated in the top-down control of visual attention (Kastner, 2004) and show similar patterns of increased activation in older adults when completing tasks such as visual search (Madden, 2007). The extent to which both declining executive function and neural compensation in ageing contribute to differences in control of visual attention remains unclear. Given that this is not the focus of the current study, we used age as the indicator for potential decline rather than characterising it through other measures such as working memory performance. Control of visual attention measured by gaze behaviour (see Kristjánsson, 2011 for discussion of the relationship between eye movements and visual attention) may, at least partially, explain age-related route learning differences.

Not all locations in an environment are equally important for route navigation. The parts of a route where a decision about the direction of travel has to be made are known as decision points (e.g. intersections), while other parts which only allow for one possible direction of travel are referred to as non-decision points. Route navigation can be conceptualised as a series of paths between decision points (Schinazi & Epstein, 2010). Objects at such decision points, i.e. landmarks, not only yield better recognition memory and recall of associated direction than objects located at non-decision points (Janzen, 2006; von Stülpnagel & Steffens, 2013), they also selectively recruit the parahippocampal gyrus (Janzen & van Turennout, 2004; Janzen & Weststeijn, 2007). Good navigators demonstrate better memory consolidation of decision point information than poor navigators (Janzen, Jansen & van Turennout, 2008).

Given the importance of decision points for successful route learning, it is not surprising that navigators pay particular attention to these locations. Using a secondary auditory probe task, Allen and Kirasic (2003) demonstrated stronger attentional engagement at areas of high navigational relevance, such as decision points. In their task, participants learned a route from a series of photographs, whilst responding to an auditory probe (a beep). Time to disengage from the primary route learning task and respond to the probe reflects the level of attentional engagement and increased at navigationally relevant locations. Hartmeyer, Grzeschik, Wolbers and Wiener (2017) replicated these findings in an ageing study using videos instead of photographs. Interestingly, the effect was similar in the younger and older age groups, even though the latter group showed marked route learning performance deficits. However, the environment used in this experiment was very simplistic, featuring empty corridors and single landmarks at decision points and turns. In view of research demonstrating that older adults have difficulty ignoring task-irrelevant stimuli (e.g. Tusch, Alperin, Holcomb & Daffner, 2016; West, 1999), it is conceivable that attentional control will be more strongly affected by age in environments which are visually more complex. If older adults directed attentional resources towards task-irrelevant stimuli, fewer resources would remain available for the primary route learning task. The disadvantage of poor resource allocation may be particularly costly for older adults considering the suggestion that their overall pool of cognitive resources may already be diminished compared to younger adults (Meulenbroek et al., 2010). Assigning already diminished attentional resources to non-task-relevant information in a complex environment would likely impact route learning performance.

In the present study, we used a paradigm similar to that of Hartmeyer et al. (2017), but we used a more visually complex environment and we tracked participants’ gaze behaviour. Our main research questions were: (1) Will previous attentional engagement findings from an auditory probe task be replicated in a complex environment? (2) Can age-related differences in route learning ability be related to differences in gaze behaviour?

The behavioural part of this study was confirmatory. We expected impaired route learning performance in our older as compared to our younger participants (c.f. Wiener et al., 2012). Moreover, we expected longer response times to the auditory probe at decision points vs. non-decision points in the younger participant group (c.f. Hartmeyer et al., 2017). If older adults were impaired in their ability to modulate engagement of attention during route learning in an information-rich environment, we expected a reduced difference in response times to the auditory probe at decision points vs. non-decision points as compared to the response time difference in younger adults.

The lack of previous research investigating how cognitive ageing affects gaze behaviour during route learning warrants an exploratory approach to the eye-tracking part of this study. If control of visual attention contributes to age-related differences in route learning, we expected systematic differences in gaze behaviour between age groups.

## Materials and methods

### Participants

Fifty-nine participants took part in the experiment. Of these, data from 13 participants had to be discarded due to technical issues with the video presentation during the experiment. Two participants also withdrew before completion of the experiment due to experiencing motion sickness and four more were discarded because they did not engage with the experiment and failed to follow instructions. Participants were screened for mild cognitive impairment using the Montreal Cognitive Assessment (MoCA; Nasreddine et al., 2005). One participant was excluded from analysis based on a cut-off score of 23 (Luis, Keegan & Mullan, 2009; Waldron-Perrine & Axelrod, 2012). Twenty younger participants (10 females; mean age 24 years; mean MoCA 28.15) and 19 older participants (10 females; mean age 73.36 years; mean MoCA 27.68) were included in the final analysis. Ethical approval was granted by Bournemouth University Research Ethics Panel and written informed consent was gained from all participants who either participated in exchange for course credits or a monetary compensation for their time.

### Design

#### Learning phase

**Fig. 1:**
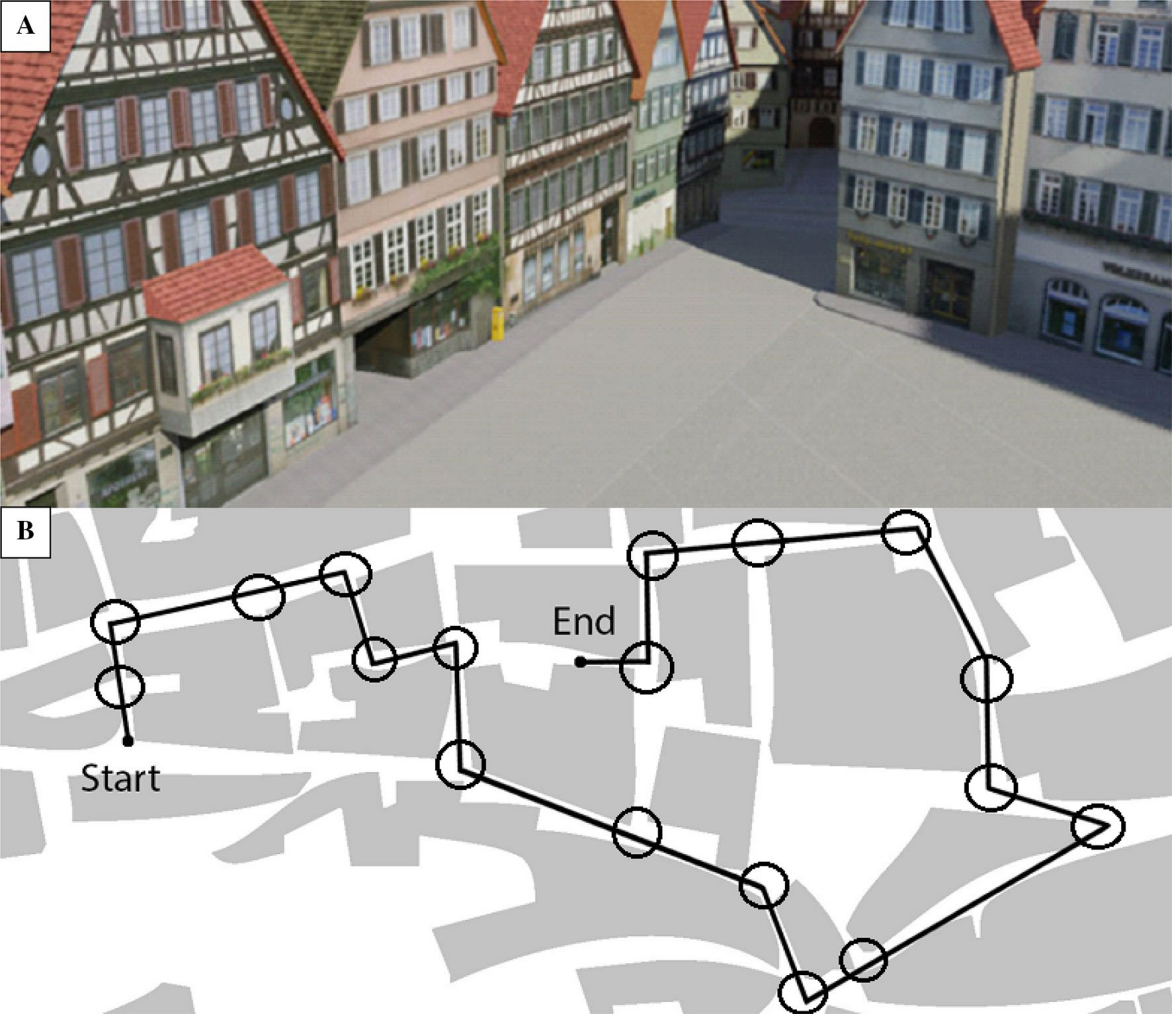
**a** A screenshot from Virtual Tübingen; **b** an overhead schematic of the route with decision points circled

Participants were passively navigated along a route through “Virtual Tübingen”, a photorealistic virtual model of Tübingen, Germany (see Fig. 1a; van Veen, Distler, Braun & Bültoff, 1998). The route, presented as a video, consisted of 18 decision points (balanced for turning directions) and was 6 min and 13 s long (see Fig. 1b). The video is available as supplementary material.

Participants were instructed to learn the route so that they would be able to reproduce it on their own. At the same time, we administered an auditory probe task as used in Allen and Kirasic (2003) and Hartmeyer et al. (2017): whilst the participants were watching the video of the route, auditory stimuli (a beep: 100 ms, square wave 1000 Hz) were presented, to which participants had to respond via key press as fast as possible. Twenty-four auditory probes were presented along the route, with their locations balanced evenly across decision points and non-decision points. The auditory probes were presented at different locations along the route in each experimental block to avoid participants predicting their onset.

#### Direction test

Participants were shown screenshots of all 18 decision points (intersections) along the route in a randomized order. Their task was to indicate the direction in which the route continued. Fifteen intersections had two possible turn directions and the rest had three. The Direction Test was the primary measure of route learning and was completed after each Learning Phase.

#### Order test

The Order Test examined participants’ knowledge of the sequence in which locations along the route were encountered. Participants were presented with two screenshots from the route next to each other and asked to indicate which location they encountered first along the route during the Learning Phase. Altogether there were 54 trials split over three image pair types. The image pair types were both decision points (18 trials), both non-decision points (18 trials) or one of each (18 trials). This task was presented at the end of the experiment and participants were not informed about the order test before it was presented, to avoid intentional changes in learning strategy to acquire sequence knowledge.

### Materials

The experiment was presented on a 102-cm screen (diagonal) with an aspect ratio of 16:9 and a resolution of 1920 × 1080 pixels. Participants sat 1 m away from the screen. Responses were recorded using a Cedrus response box (RB-740, Cedrus, San Pedro, USA). Auditory stimuli were presented via speakers using an external sound card (ASIO M-Track Plus, M-Audio, Cumberland, USA). Eye movements were recorded using an Eyelink II (SR Research) head mounted eye-tracker at a rate of 500hz. Calibration used a 9-point grid, and a drift correction was performed before every phase of the experiment.

### Procedure

The experiment took approximately 1 h and was divided into several phases. Before the experiment began, participants completed a short practice session to familiarise themselves with the tasks and to check their understanding of the instructions. This session used a short two-intersection route through a different part of Virtual Tübingen and included response to auditory stimuli. There was also a short Direction Test with two trials. There was no Order Test in the practice session to ensure that participants remained unaware of this task. The main experiment comprised of two experimental blocks, each containing a Learning Phase followed by the Direction Test. Each block used the same route. The Order Test was administered once at the end of the experiment.

### Eye-tracking measures

Given the lack of previous research linking gaze behaviour, ageing and route learning, we took an exploratory approach to analysing eye-tracking measures during route learning. As in Dowiasch et al. (2015), we compared saccade amplitude, peak velocity, average velocity and frequency (number of saccades per second) between age groups for eye-tracking data from the learning phase using analysis of variance (ANOVA). We did the same for the Direction Test with the addition of average fixation duration. We decided not to analyse gaze behaviour for the Order Test as the angular size of the stimuli was relatively small (to present two scenes at once).

Due to the dynamic nature of the video stimuli used in the Learning Phase and the complex spatial scenery, it was difficult to relate gaze behaviour to specific environmental features. Regions of interest analysis as described in Allen and Kirasic (2003) are not applicable to video stimuli [see also Caldara and Miellet (2011) for a discussion on the limitations of regions of interest analyses]. An alternative option would be a frame-by-frame analysis of gaze behaviour during the Learning Phase. This, however, would have been very labour intensive, for example, Anderson et al. (2012) report approximately 31 h of hand coding per hour of video data. Consequently, such methods are typically applied only to samples much smaller than in the current study (e.g. Hollands, Patla, & Vickers, 2002; Imai, Moore, Raphan, & Cohen, 2001). We opted for two other analyses for the Learning Phase.

First, we developed a new measure: gaze dispersion. Gaze dispersion is calculated as the average distance of all gaze points, within a specified time window of the video, from the centre of gravity of those points. High gaze dispersion values mean that participants’ gaze is widely distributed across the stimulus and can therefore be described as more exploratory, while smaller dispersion values indicate more spatially focused and less exploratory gaze behaviour. We calculated gaze dispersion in a 1000 ms moving time window, with a 500 ms overlap between each successive window, and analysed how gaze dispersion changed during the last 5 s of approaching a decision point. We used a linear mixed effect model (LME) analysis to investigate whether time until reaching a decision point could predict gaze dispersion in younger and older participants.

Second, we analysed the effect of route learning between blocks 1 and 2 on gaze bias at decision points. Specifically, we were interested whether the likelihood of gaze being directed towards the correct direction of travel changed between the first and the second viewing of the route. Any increase in the likelihood that the correct path option was attended to would reflect learning of the route. The gaze bias analysis is spatially sensitive to where gaze is directed in the environment, while the gaze dispersion measure is temporally informative.

The Direction Test comprised of static stimuli (screenshots) which allowed us to use iMap4 (Lao, Miellet, Pernet, Sokhn & Caldara, 2017) to analyse gaze behaviour. Specifically, we analysed whether age systematically affected what parts of the stimuli participants looked at when recalling route directions at decision points. iMap4 is a MATLAB open source toolbox implementing a pixel-wise linear mixed model approach for statistical fixation mapping of eye movement data and nonparametric tests based on resampling to assess statistical significance.

## Results

### Behavioural

#### Auditory probe task

Responses from one participant were not collected due to user error so they were excluded from the analysis.

A repeated measures ANOVA on response times with a between group factor of age group (young, old) and within group factors of block number (1, 2) and section type (DP or NDP) revealed main effects of age group [*F*(1, 36) = 11.02, *p* = 0.002, *ηp*^2^ = 0.234] and section type [*F*(1,36) = 52.49, *p* < 0.001, *ηp*^2^ = 0.593], but no main effect of block [*F*(1,36) = 0.87, *p* = 0.358, *ηp*^2^ = 0.024]. Younger adults (403.52 ms) responded faster to the auditory probes than older adults (487.51 ms). Probes at non-decision points (425.44 ms) were responded to faster than at decision points (465.29 ms). Only the age group × section type interaction was significant [*F*(1,36) = 8.06, *p* = 0.007, *ηp*^2^ = 0.183].

**Fig. 2:**
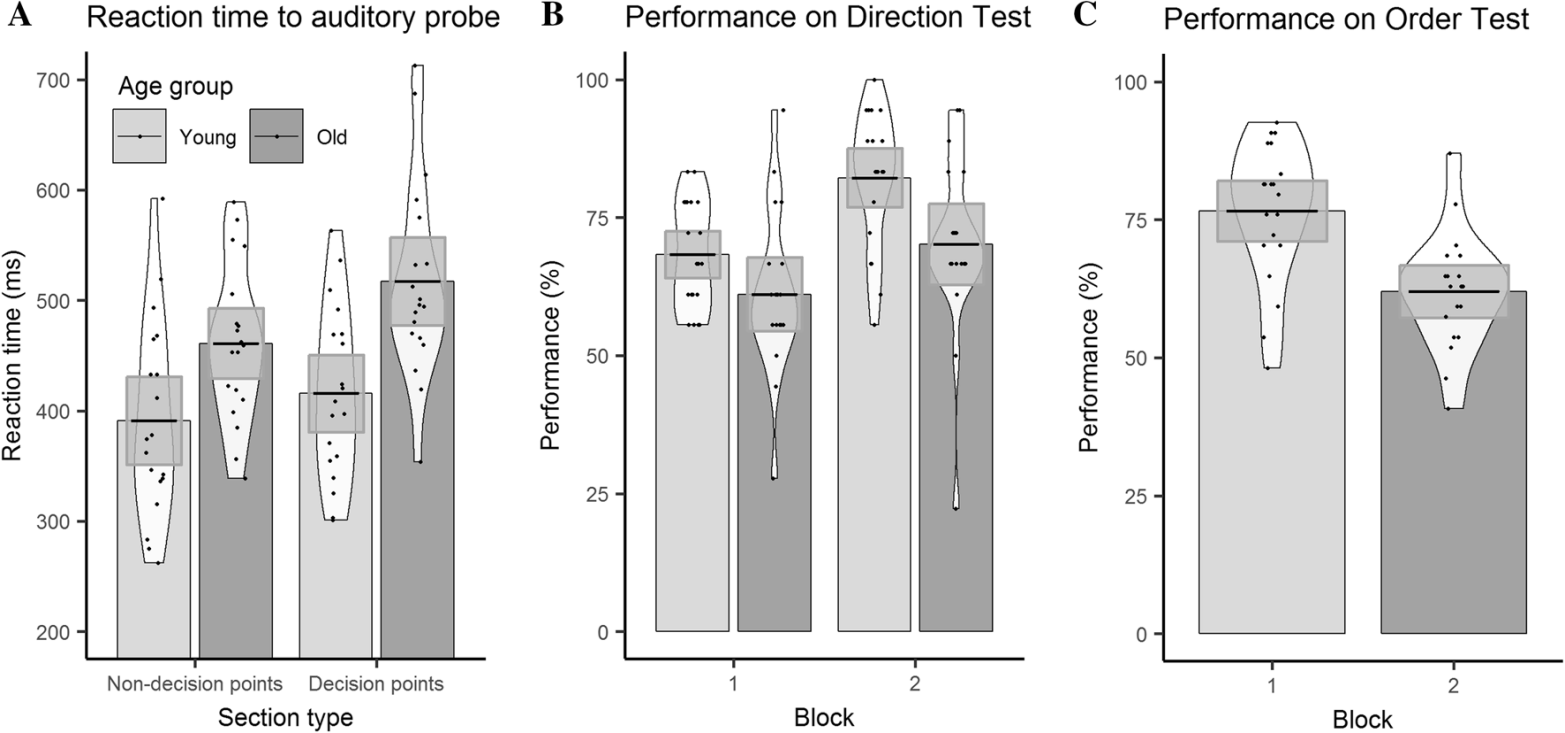
Graphs include means, 95% confidence intervals and density profiles. **a** Reaction times of younger and older participants to the auditory probe at decision and non-decision points; **b** performance of younger and older participants on the Direction Test over blocks 1 and 2; **c** performance of younger and older participants on the Order Test

To investigate this interaction, which indicates that the size of the section type effect is larger for the older participant group compared to the younger group, the data were split by age group and paired *t* tests were performed for section type. Both younger adults [*t*(18) = 5.57, *p* < 0.001, *d* = 0.22] and older adults [*t*(18) = 5.44, *p* < 0.001, *d* = 0.43] were significantly faster at responding to the auditory probe at non-decision points (younger = 391.02 ms, older = 461.03 ms) than at decision points (younger = 415.71 ms, older = 517.11 ms; see Fig. 2a).

#### Direction test

A repeated measures ANOVA on performance with a between group factor of age group (young, old) and a within group factor of block number (1, 2) revealed main effects of age group [*F*(1, 37) = 6.12, *p* < 0.001, *ηp*^2^ = 0.142] and block number [*F*(1, 37) = 39.54, *p* < 0.001, *ηp*^2^ = 0.517]. Younger participants (75.28%) performed better than older participants (65.64%) and performance on block 2 (76.35%) was better than on block 1 (64.81%; see Fig. 2b). There was no significant interaction.

#### Order test

A repeated measures ANOVA on performance with a between group factor of age group (young, old) and a within group factor of pair type (decision points, non-decision points, mixed) revealed a main effect of age group [*F*(1, 37 = 15.30), *p* < 0.001, *ηp*^2^ = 0.293] but no main effect of pair type [*F*(1, 37) = 2.07, *p* = 0.159, *ηp*^2^ = 0.053]. Younger adults (76.57%) performed better than older adults (61.99%; see Fig. 2c). There was no significant interaction.

### Eye-tracking

One older participant had a visual disorder that prevented eye-tracking. There were also calibration errors for two older participants during the Learning Phase; these participants were not included in the analysis of gaze behaviour during the Learning Phase.

#### Learning phase

##### Saccade parameters

Separate repeated measures ANOVAs were conducted for saccade amplitude, frequency, peak velocity and average velocity with a between group factor of age group and a within group factor of block. There were no significant effects of age group or block on any of the saccade parameters (see Table 1 for the age differences’) and no significant interactions.

**Table 1:** Means and ANOVA results for saccade parameters between younger and older adults from the Learning Phase

Saccade parameter	Mean (SD) young	Mean (SD) old	*f* value	*p* value	*ηp*^2^
Saccade amplitude (°va)	6.58 (1.30)	6.24 (1.16)	0.65	0.425	0.012
Saccade peak velocity (°/s)	251.71 (49.99)	249.10 (37.72)	0.03	0.871	< 0.001
Saccade average velocity (°/s)	139.82 (17.75)	130.66 (15.78)	2.57	0.118	0.070
Saccade frequency (/s)	2.48 (0.45)	2.71 (0.43)	2.44	0.128	0.067

Given the exploratory nature of this study, the lack of theoretically motivated prior expectations, and the sensitivity of Bayes factors to these prior expectations (Liu & Aitkin, 2008; Morey, Romejin & Rouder, 2016), we decided against using Bayes factors to support the null hypothesis of no differences between age groups. Instead, we used bootstrapped *t* tests (5000 resamplings) to take into account the real distribution of our data. First, data were centred to the mean to ensure H0. Then a bootstrapped *t* value was computed after random sampling of 20 centred data points with replacement. This procedure was repeated 5000 times. The 5000 bootstrapped *t* values were then ordered, and 0.025 and 0.975 bounds determined. Finally, the observed *t* value was compared to the bootstrapped bounds to assess significance. Here, there was no significant effect of age on amplitude (*t*-obs = 0.83; 95% CI [0.03, 2.37]; *p* = 0.404), peak velocity (*t*-obs = 0.19; 95% CI [0.03, 2.30], *p* = 0.847), average velocity (*t*-obs = 1.64; 95% CI [0.03, 2.32], *p* = 0.164) or frequency (*t*-obs = 1.57; 95% CI [0.04, 2.43], *p* = 0.123).

##### Gaze dispersion

Using the raw data samples, gaze dispersion for every 1000 ms of the Learning Phase was calculated with a 500 ms overlap for each time subsequent time window. We analysed gaze dispersion for the last 5000 ms during the approach of decision points during learning, with 0 ms being the arrival at the intersection, but before a turn would be initiated.

We ran a linear mixed effects model (LME) analysis for dispersion of gaze using the lme4 package (version 1.1-14; Bates, Machler, Bolker & Walker, 2015) in R (R Core Team, 2017). Fixed effects were approach time to intersection (continuous, 5000–0 ms, centred around 0), block number (factor, 1 or 2, centred using sum contrast coding) and age group (factor, young or old, centred using sum contrast coding). Random effects were subject and intersection. We started with an intercept only model and added random by-subject and by-intersection slopes for fixed effects one by one (starting with those that accounted for the most variance) and then added interactions between random slopes. Each random slope or interaction was included only if it significantly improved the model.

**Fig. 3:**
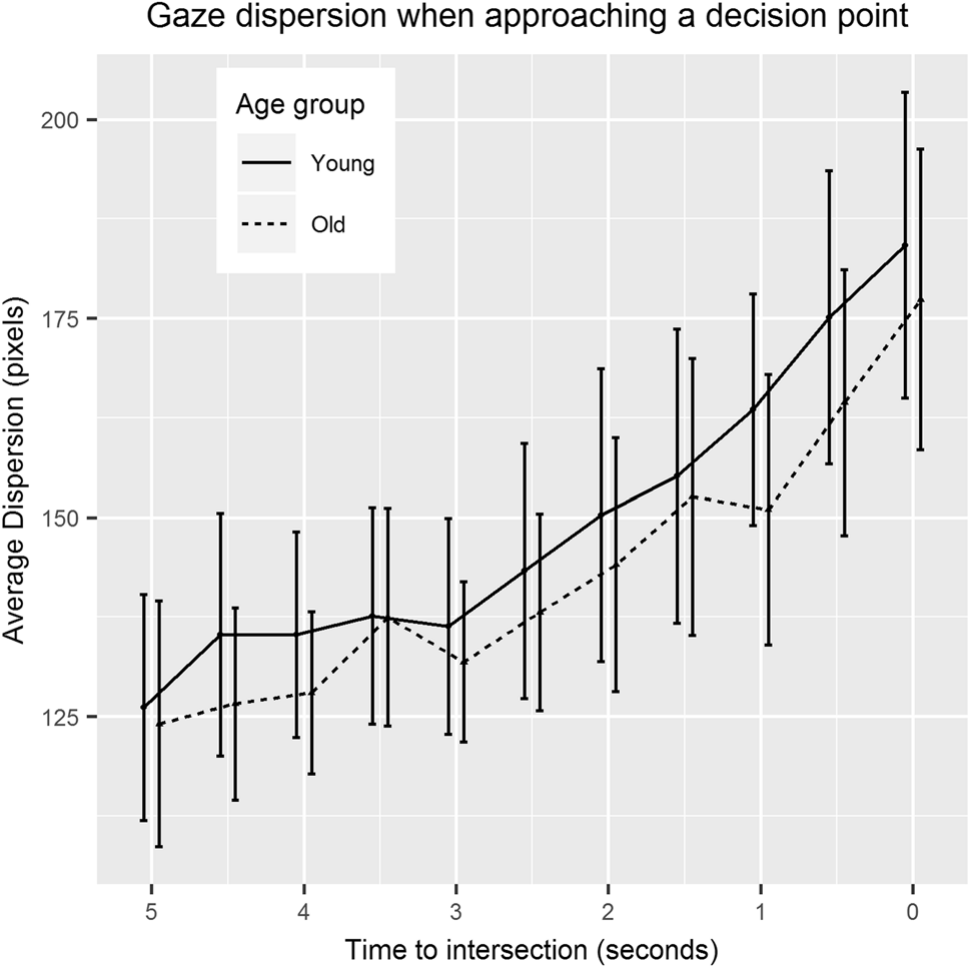
Dispersion of gaze on the five second approach to an intersection for younger and older participants

Estimates, standard errors and *t* values for the final model are reported in Table 2 and show that approach time to an intersection is a significant predictor of gaze dispersion (see Fig. 3), whilst age group and block do not have a significant effect. There were no significant interactions.

**Table 2:** Coefficients from LME analysis

Fixed effect on dispersion of gaze (number of pixels)	Estimate	Std. error	*t* value
Intercept	146.24	7.77	18.82^a^
Approach time	10.10	2.18	4.63^a^
Age group	3.05	4.84	0.63
Block	2.42	1.72	1.41

^a^Significant *t* values (|*t*| ≥ 1.96)

##### Gaze bias

We used the raw data samples to calculate gaze bias during the last 5000 ms before entering a decision point. To do this, the screen was split into three equal regions to represent left, straight and right turns. Gaze bias is defined as the percentage of samples which were located in the region that corresponded with the correct direction of travel.

**Fig. 4:**
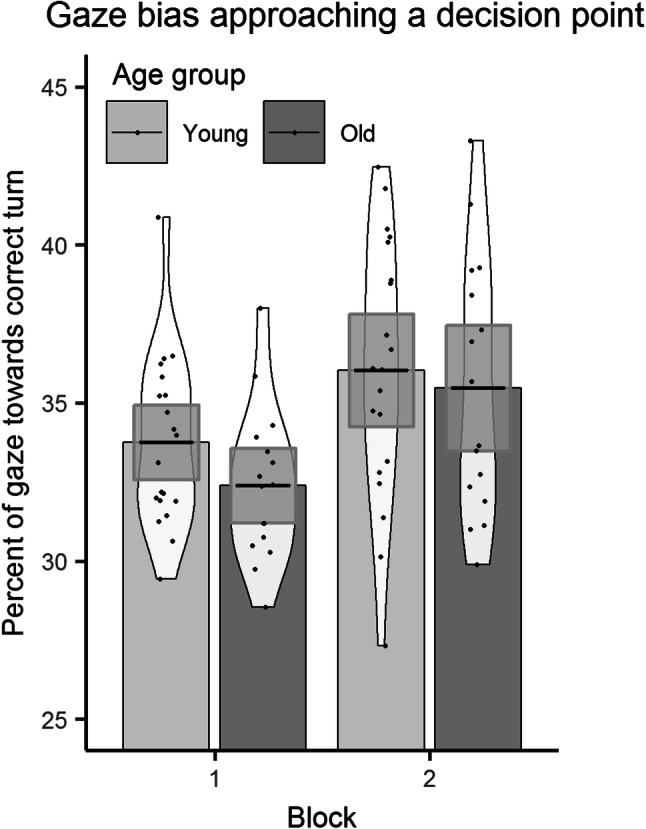
Gaze bias towards correct direction in the learning phase across blocks for younger and older participants

A repeated measures ANOVA on gaze bias with a between group factor of age group (young, old) and a within group factor of block number (1, 2) revealed a main effect of block [*F*(1, 34) = 11.31, *p* = 0.002, *ηp*^2^ = 0.250] and no main effect of age group [*F*(1, 34) = 1.39, *p* = 0.247, *ηp*^2^ = 0.039]. Gaze bias was higher during block 2 (35.79%) than during block 1 (33.15%; see Fig. 4). There was no significant interaction.

##### Gaze behaviour and route learning performance

To investigate whether the gaze dispersion and gaze bias measures were in fact predictive of route learning performance, we ran a multiple regression analysis with gaze bias and gaze dispersion as predictor variables and performance in the behavioural direction test as the outcome variable. We included block as an interaction term for gaze bias. This was motivated by gaze bias being a measure of learning and thus is at chance level for block 1 since this was the first time participants had seen the route. A significant regression equation was found [*F*(3, 68) = 5.25, *p* = 0.003] with an *R*^2^ of 0.15. Gaze dispersion was a significant predictor of test phase performance [*b* = 0.04, *t*(68) = 2.36, *p* = 0.02]. Gaze bias was also a significant predictor of direction test performance, but only in block 2 [block 1: *b* = 0.02, *t*(68) = 0.89, *p* = 0.38; block 2: *b* = 0.07, *t*(68) = 3.067, *p* = 0.003].

#### Direction test

##### Saccade and fixation parameters

Separate repeated measures ANOVAs were conducted for saccade amplitude, frequency, peak velocity, average velocity and for average fixation duration with a between group factor of age group and a within group factor of block. Block only rendered a significant effect on saccade frequency [*F*(1, 37) = 17.58, *p* < 0.001, *ηp*^2^ = 0.322] and the main effect of age group was only significant for saccade average velocity [*F*(1, 37) = 6.68, *p* = 0.014, *ηp*^2^ = 0.153] (see Table 3 for the age differences’). There were no significant interactions.

**Table 3:** Means and ANOVA results for saccade and fixation parameters between younger and older adults for the test phase

Gaze parameter	Mean (SD) younger	Mean (SD) old	*f* value	*p* value	*ηp*^2^
Saccade amplitude (°va)	8.65 (1.17)	8.11 (1.30)	1.44	0.237	0.038
Saccade peak velocity (°/s)	292.44 (41.01)	285.65 (42.83)	0.17	0.69	0.005
Saccade average velocity (°/s)	166.48 (16.28)	151.31 (17.86)	6.68	0.014	0.153
Saccade frequency (/s)	3.61 (0.31)	3.44 (0.50)	1.46	0.235	0.038
Average fixation duration (ms)	258.05 (25.61)	259.40 (31.34)	0.01	0.923	> 0.001

Bootstrapped *t* tests showed a significant effect of age on average velocity (*t*-obs = 2.77; 95% CI [0.03, 2.34], *p* = 0.008) and no significant effects of age on amplitude (*t*-obs = 1.38; 95% CI [0.03, 2.31]; *p* = 0.168), peak velocity (*t*-obs = 0.51; 95% CI [0.04, 2.34], *p* = 0.617), frequency (*t*-obs = 1.24; 95% CI [0.03, 2.59], *p* = 0.238) or fixation duration (*t*-obs = 0.15; 95% CI [0.03, 2.36], *p* = 0.888).

##### iMap

iMap 4 (Lao et al., 2017) was used to examine the regions in each test stimulus to which participants directed their gaze. Participant and test stimulus were included as random effects, and fixed effects were age group and block. The model showed no significant effect of age group or block on location of gaze during the test phase.

## Discussion

The overall aim of this study was to investigate the potential contribution of control of visual attention and attentional engagement to age-related changes in route learning ability. We compared an older and younger participant group using a standard route learning paradigm with eye-tracking and an auditory probe task. We found an age-related performance deficit in tests of route knowledge. Both younger and older participants were slower to respond to the auditory probe at decision points as compared to non-decision points and this slowing was statistically larger for the older participant group. We report a significant increase in gaze dispersion on the approach to an intersection and an effect of learning on gaze bias towards the correct path option. Finally, there were no age differences in both the learning phase and the direction test phase on several gaze measures, including general saccade parameters (other than average velocity in the test phase), gaze dispersion, gaze bias and iMap analysis.

### Route learning performance and attentional engagement

Older adults performed worse than younger adults on both the Direction Test and the Order Test, which is consistent with earlier navigation studies (Hartmeyer et al., 2017; Head & Isom, 2010; Wiener et al., 2012). Difficulty in recalling directions at intersections for older adults suggests age-related deficits in place–response associations (see Strickrodt, O’Malley & Wiener, 2015). Zhong and Moffat (2016) suggest this place-response deficit is due to older adults expending more cognitive resources on the encoding of landmark/place information than on the binding of this information to a direction. In relation to the results in the Order Test, a lack of knowledge about the relative locations of intersections along the route in older adults indicates impairment in place–place associations (see Strickrodt et al., 2015). Place–place associations are important, as navigation of an environmental scale space may not be encoded in a single reference frame, but in several smaller scale reference frames which are linked by proximity and are switched between as the environment is traversed (Meilinger, 2008; Schinazi & Epstein, 2010; Wolbers & Wiener, 2014). This allows navigators to form expectations of next encounters and plan responses accordingly (Schölkopf & Mallot, 1995), a task in which older adults are impaired (Salthouse & Siedlecki, 2007).

Task focused engagement of attentional resources is reflected in the time taken to disengage and respond to a secondary auditory probe task (Posner & Boies, 1971). We show slower response times to probes presented at decision points as compared to non-decision points, suggesting engagement of more attentional resources to the navigation task at locations important for route learning (Allen & Kirasic, 2003). This effect is present for both younger and older participants. We also find an interaction between age group and probe location. However, this interaction is difficult to interpret as older adults typically show slower cognitive processing (Salthouse, 2000; Waters & Caplan, 2005), which could be amplified by the need to disengage more resources from the primary task. Our results replicate findings from Hartmeyer et al. (2017) in a complex environment, providing more evidence that route learning deficits in older adults cannot be explained by changes in the deployment of attentional resources.

### Gaze behaviour and route learning

First, we introduced gaze dispersion as a new eye-tracking measure. We found that dispersion increased during the approach to a decision point. One possible explanation for this is that the visual features of the environment may be driving the dispersion effect. Viewers of a spatial scene will preferentially direct gaze to areas with the longest line of sight (Wiener et al., 2012). In the case of a non-decision point location, this would usually be the path of travel and thus gaze would be focused here. In the case of a decision point, where multiple paths are available, gaze may be split between path options, thus leading to an increase in gaze dispersion.

Alternatively, an increase in gaze dispersion may be task driven. Given that navigators selectively encode information at decision points (Janzen, 2006; Janzen, Jansen & van Turennout, 2008); an increase in dispersion could reflect wider exploration of the environment to obtain more information about that specific navigationally relevant location. Second, we analysed gaze bias at decision points during route learning. We found that when participants saw the route for the second time, they were more likely to look at the correct direction of travel during the 5 s approach of decision points. This is in line with previous accounts of gaze bias for eventually chosen path options in a spatial task (Wiener et al., 2012).

The results from these two gaze measures fit with the spatial decision making framework during navigation recently reported by Brunyé, Gardony, Holmes and Taylor (2018). They suggest that decision making occurs before a decision point is entered. In their study, participants could request additional information about route direction (in the form of a beacon) and were most likely to request information during the 5 s before entering a decision point. In our study, gaze dispersion begins to increase around 5 s before an intersection is entered. We believe this reflects the acquisition of information during the approach of a decision point through wider visual exploration to aid decision making when later recalling the route. When participants approached an intersection during the second training phase, they showed a gaze bias towards the correct direction of travel. These results nicely reflect the advanced spatial decision making described in Brunyé et al. (2018). Specifically, the gaze bias suggests that our participants were able to predict the direction of travel before reaching the decision point, most likely based on information obtained through the increased visual exploration of the environment at decision points in the previous exposure.

Overall, the change in gaze dispersion and in gaze bias as a function of learning demonstrates that eye-tracking measures are sensitive to the spatial environment and the route learning task. This is further corroborated by the regression analysis showing that both of these measures predicted learning performance.

### Gaze behaviour and ageing

We compared older and younger participants on several eye movement parameters during route learning and the recall of directions at decision points. First, we focused on general gaze patterns during the learning phase. For the vast majority of measures, there were no differences between age groups, which was surprising, given evidence from Dowiasch et al. (2015), who reported age differences in general gaze parameters. The bootstrapped *t* test analysis demonstrated that our observed comparisons fall well within the distribution of *t* values when comparing groups without differences, suggesting our results represent no difference between age groups as opposed to a type 2 error. Further, we found no difference between age groups in gaze dispersion, whilst learning a route, gaze bias in response to learning or in the distribution of gaze locations in the Direction Test. We found a difference in average saccade velocity during the test phase, however, in view of the overwhelming similarity in the other measures of eye movements; we do not attribute too much meaning to this. Our findings suggest that age-related performance differences in route learning are not reflected in gaze behaviour.

A possible explanation for the differences in results between our study and the study by Dowiasch et al. (2015) comes from the fact that participants in their study were actively locomoting through the environment, while our participants were passively transported along the route. It is conceivable that age-related differences in postural control (Jimenez et al., 2016), control of locomotion and steering cause differences in gaze behaviour. For example, Uiga et al. (2015) suggest that older adults focus more on the lower portion of the visual field, potentially because they are more afraid of falling and therefore closely monitor the space just in front of them. Here we used Montello’s (in Shah & Miyake, 2005) definition of navigation which is comprised of two components: wayfinding and locomotion. While wayfinding refers to the memory and decision making processes involved in navigation, locomotion is about coordinating movement in the local environment. As we aimed to isolate the cognitive processes involved in the route learning and spatial decision making, we decided to use passive navigation. In other words, we eliminated age effects that related to steering and other aspects of locomotory control. However, it would be interesting if future work would investigate how age-related changes in gaze behaviour related to locomotion might interact with route navigation ability.

Given the similarity of eye movement parameters between age groups, we suggest little, if any, task-related difference in oculomotor behaviour between age groups. This is in line with Pratt, Abrams and Chasteen (1997) who report a simple saccade to target task where they conclude that older adults produce saccades in fundamentally the same way as younger adults, and in follow-up work (Abrams, Pratt & Chasteen, 1998; Pratt, Dodd & Welsh, 2006), demonstrate equivalence between age groups on many basic eye movement parameters. Age differences in eye movements in other work can be attributed to a cognitively driven difference such as using different strategies or cues to solve a task. Thus, results from our study showing equivalence in oculomotor behaviour between age groups in route learning indicate that both age groups use similar visual strategies during route learning. As discussed earlier, performance differences could then be explained by associative learning deficits in stimulus–response associations instead of differences in oculomotor control.

## Conclusion

In summary, we have replicated previous findings showing that attentional resources are dedicated to decision points in route navigation and that this process is not affected by ageing. Further, the general control of visual attention does not differ between older and younger participants when learning and recalling route information. Taken together, we conclude that route learning deficits in typically aged adults are not reflected in changes in attentional engagement or by general changes in visual attention. More specific gaze measures may be more sensitive in identifying precise artefacts of visual attention which could be used to further investigate changes in ageing. Our current and future work involves further development of spatially sensitive measures appropriate for dynamic stimuli, focussing on the environmental content of gaze location. Finally, we demonstrate a change in gaze bias in response to route learning and find that gaze dispersion is sensitive to changes in the spatial stimuli, both of which predict route learning performance.

## Electronic supplementary material

Below is the link to the electronic supplementary material.

Supplementary material 1 (XVD 221145 KB)

## Data Availability

The datasets analysed during the current study are available from the corresponding author on reasonable request.
